# Large-Scale Studies on Antimicrobial Resistance and Molecular Characterization of Escherichia coli from Food Animals in Developed Areas of Eastern China

**DOI:** 10.1128/spectrum.02015-22

**Published:** 2022-08-11

**Authors:** Jiangang Ma, Wei Zhou, Jing Wu, Xiaofeng Liu, Jiahui Lin, Xiaofeng Ji, Hui Lin, Jianmei Wang, Han Jiang, Qianjin Zhou, Guoping Zhao, Hua Yang, Biao Tang

**Affiliations:** a State Key Laboratory for Managing Biotic and Chemical Threats to the Quality and Safety of Agro-products, Institute of Agro-product Safety and Nutrition, Zhejiang Academy of Agricultural Sciences, Hangzhou, Zhejiang, China; b Zhejiang Provincial Center for Animal Disease Prevention and Control, Hangzhou Zhejiang, China; c College of Food Science and Technology, Zhejiang University of Technology, Hangzhou, Zhejiang, China; d Key Laboratory of Marine Food Quality and Hazard Controlling Technology of Zhejiang Province, China Jiliang University, Hangzhou, Zhejiang, China; e School of Marine Science, Ningbo University, Ningbo, Zhejiang, China; f CAS Key Laboratory of Synthetic Biology, Institute of Plant Physiology and Ecology, Shanghai Institutes for Biological Sciences, Chinese Academy of Sciences, Shanghai, China; University of Maryland Eastern Shore

**Keywords:** *Escherichia coli*, antimicrobial resistance, antimicrobial resistance gene, food animal, heavy metal

## Abstract

Widely distributed multidrug-resistant (MDR) bacteria threaten animals and human health. Nevertheless, few antimicrobial resistance (AMR) surveys of large-scale animal-derived bacteria have been explored. Here, 1,468 (97.54%) Escherichia coli strains were isolated from 1,505 pig (1,060) and chicken (445) anal swab samples from 11 cities in Zhejiang Province, China, in 2020. These isolates had a high resistance to tetracycline (92.92%), sulfisoxazole (93.05%), florfenicol (83.11%), and ampicillin (78.27%). More than 88.68% of the strains were MDR bacteria. A low AMR ratio to the “last-resort” antimicrobials tigecycline (0.75%), colistin (1.36%), and meropenem (0.75%) were found. The AMR of E. coli from pigs was higher than that of chickens. Eighteen strains among 31 MDR strains that were resistant to “last-resort” antimicrobials could transfer the AMR genes (*mcr-1*, *tet*(X), and *bla*_NDM_) to the recipient strain J53, which confer colistin, tigecycline, and carbapenem resistance, respectively. The homology among *mcr-1*-carrying isolates was relatively high, and the sequence types were mainly ST5529, ST101, and ST354, while the homology of isolates harboring *tet*(X4) and *bla*_NDM-5_ genes were different. The *mcr-1*, *bla*_NDM-5_, and *tet*(X4) genes in strains LS45, JH51, and TZ118 were identified on the Incl2, IncHI2, and IncX1 plasmids, respectively. Moreover, *tet*(A), *sul2*, *floR,* and *bla*_TEM-1B_ were the most common ARGs in 31 strains. Additionally, the heavy metals copper and zinc had a significant correlation with amoxicillin/clavulanate and tetracycline resistance. Controlling the movement of animals between cities and reducing the use of antimicrobials are effective methods to reduce the threat of AMR bacteria.

**IMPORTANCE** Pigs and chickens are the most common food animals that are the important vectors for spreading antimicrobial-resistant pathogens among animals and humans. Limited systematic AMR monitoring of these food animal origin bacteria had been reported, especially in developed areas of China. Our study provides a comprehensive and systematic study of AMR in Escherichia coli from eastern China. The AMR of E. coli strains among the animals or cities has statistically significant differences. Moreover, the *mcr-1*, *tet*(X4), and *bla*_NDM-5_ genes, considered resistant to the last line of AMR, were identified in part of farms. The transferability and the prevalence of these AMR strains were intensively studied. Our monitoring is comparable to human clinical research and has an essential reference for public health safety. These findings will provide early warning for AMR strains and guide the clinical use of antibiotics to control the spread of antibiotic resistance.

## INTRODUCTION

Antimicrobials are widely used in animals and humans to treat or prevent diseases caused by various bacteria, and they have saved countless lives since they were discovered (1, 2). Unfortunately, antimicrobial resistance (AMR) is increasingly being discovered worldwide (3). The emergence of multidrug-resistant (MDR) bacteria severely affects the health of animals, adversely affecting the economics of the livestock industry (4, 5). Meanwhile, it also causes significant threats to patients because MDR bacteria affecting humans are closely associated with animal sources (6, 7). In particular, “last-resort” antimicrobial resistance genes (ARGs) are continually emerging, such as the *bla*_NDM_ (8), *mcr* (9), and *tet*(X) families (10, 11), in Gram-negative bacilli.

Escherichia coli is one of the most common Gram-negative opportunistic bacteria in animals and humans. It is the most frequently identified microorganism in bloodstream infections, and MDR strains have increased mortality. Moreover, E. coli is a reservoir and melting pot for AMR genes and mobile elements (12). It is a commensal bacterium in both animals and humans that can be transferred between animals and humans. Hence, E. coli is often used as a sensitive indicator for the surveillance and spread of AMR among pathogens (13). Food animals are an important medium for the development and spread of AMR among bacteria.

Monitoring the AMR of bacteria from food animals has significant implications for the treatment of bacterial infections and the prevention of ARG spread (14–16). The epidemiology and antimicrobial susceptibility of clinical E. coli are continuously monitored in China. The higher resistance rates against several antimicrobials of E. coli isolated from hospitals were observed in 2017 and 2018 (17, 18). The first-line AMR rates of E. coli from western China were higher than those in eastern China (19, 20). However, there are limited systematic monitoring reports about the animal origin of resistant bacteria, and most studies surveyed a few regions randomly and without genomic characterization (15).

Therefore, we investigated a large-scale study of the AMR of E. coli isolated from pig and chicken farms in each city in Zhejiang Province, which is one of the most developed provinces in China. Meanwhile, we analyzed the AMR genes and the genetic contexts for strain resistance against the “last-resort” antimicrobials. The results of this study provided the prevalence of AMR and the mechanisms of ARG transfer between strains.

## RESULTS

### Isolation and AMR characteristics of the E. coli isolates.

**FIG 1:**
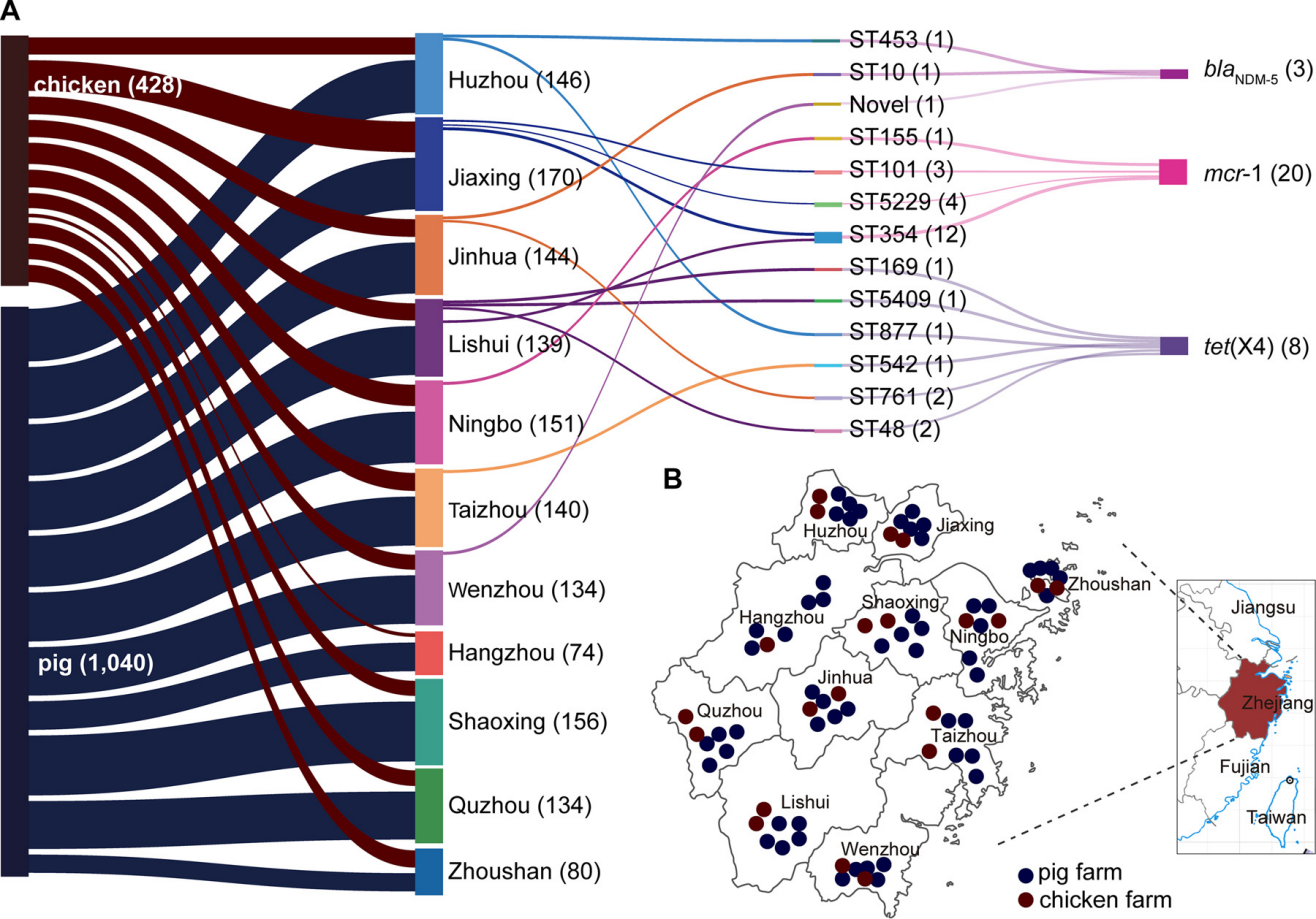
Information on the strains resistant to “last-resort” antimicrobials in this study. (A) The relationship of the resistant strains from cities and origins with the ST types. The strain numbers are marked behind the information with parentheses. (B) The distribution of these strains isolated from pigs and chickens in cities in eastern China.

**FIG 2:**
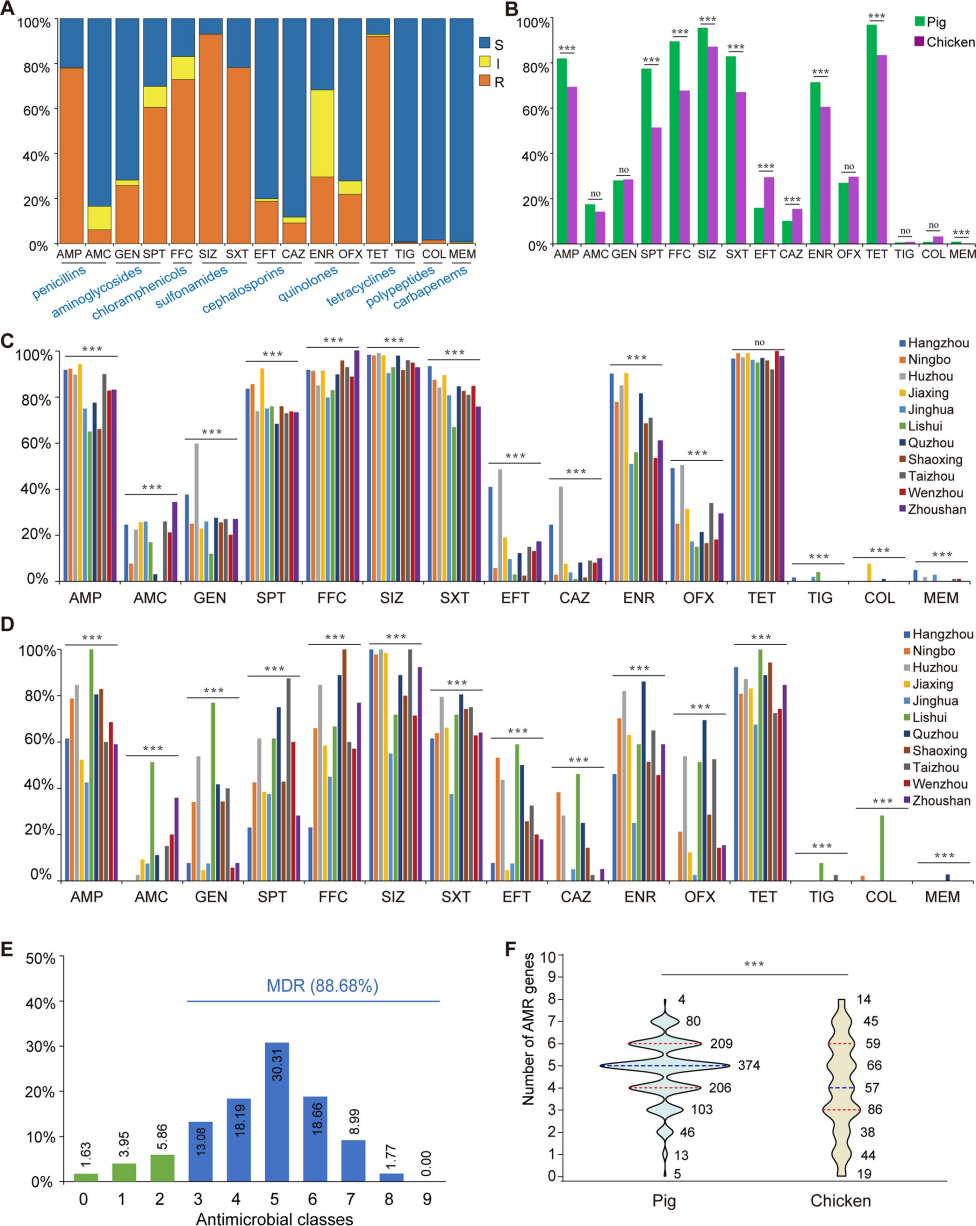
The antimicrobial-resistance phenotypes of the 1,468 E. coli from pigs and chickens in Zhejiang Province. (A) The column chart of antibiotic resistance rates of E. coli isolates. The blue and orange shading represents resistant (R) and intermediate (I) rates that were incorporated into nonsusceptible (NS). The gray shading represents the susceptible (S) rate. (B) The difference in resistance rates for the isolates from pigs and chickens among 15 antimicrobials. ***, *P* < 0.001; no, no significant difference between the two groups (*P* > 0.05). (C) The resistance rates of isolates from pigs in 11 cities. (D) The resistance rates of isolates from chickens in different cities. The blue, orange, gray, yellow, light blue, green, dark blue, brown, dark gray, red, and purple represent Hangzhou, Ningbo, Huzhou, Jiaxing, Jinghua, Lishui, Quzhou, Shaoxing, Taizhou, Wenzhou, and Zhoushan, respectively. (E) Prevalence of multidrug resistance among 1,468 E. coli isolates. The isolates’ resistance to three or more different types of antimicrobials was classified as MDR, and the rate is shown in blue. The non-MDRs are shown in green. (F) Differences in the MDR rate between the strains from pigs and chickens. The blue dotted line represents the median, and the red dotted lines represent the quartiles.

A total of 1,468 (97.54%) E. coli strains were isolated from 1,505 pigs (1,060) and chickens (445) in different cities of Zhejiang Province (Fig. 1). This survey covers pig and chicken farms in each city (Table 1). These isolates had a high resistance to tetracycline (TET, 92.92%), sulfisoxazole (SIZ, 93.05%), florfenicol (FFC, 83.11%), ampicillin (AMP, 78.27%), sulfamethoxazole/trimethoprim (SXT, 78.27%), spectinomycin (SPT, 69.82%) and enrofloxacin (ENR, 68.26%). They had relatively lower resistance to gentamicin (GEN, 28.20%), ofloxacin (OFX, 27.79%), ceftiofur (CEF, 19.89%), amoxicillin/clavulanate (AMC, 16.55%), ceftazidime (CAZ, 11.72%), colistin (COL, 1.36%), tigecycline (TIG, 0.75%) and meropenem (MEM, 0.75%) (Fig. 2A). There were 3 intermediate-TIG strains and 8 intermediate-MEM strains among the resistant strains.

**TABLE 1:** The number of E. coli strains collected from different animals and cities

City	Pig			Chicken		
	Samples	Isolates	Prevalence	Samples	Isolates	Prevalence
Hangzhou	65	61	93.85%	15	13	86.67%
Ningbo	105	104	99.05%	50	47	94.00%
Huzhou	110	107	97.27%	40	39	97.50%
Jiaxing	105	105	100.00%	65	65	100.00%
Jinhua	105	104	99.05%	40	40	100.00%
Lishui	100	100	100.00%	40	39	97.50%
Quzhou	100	98	98.00%	40	36	90.00%
Shaoxing	125	121	96.80%	35	35	100.00%
Taizhou	100	100	100.00%	40	40	100.00%
Wenzhou	100	99	99.00%	40	35	87.50%
Zhoushan	45	41	91.11%	40	39	97.50%
Total	1,060	1,040	98.11%	445	428	96.18%

The rates of AMR for the antimicrobials against the isolates from pigs and chickens were as follows: AMP (81.92% and 69.39%), AMC (17.50% and 14.25%), GEN (28.08% and 28.50%), SPT (77.40% and 51.40%), TET (96.83% and 83.41%), FFC (89.42% and 67.76%), SIZ (95.48% and 87.15%), STX (82.88% and 67.06%), CEF (15.96% and 29.44%), CAZ (10.19% and 15.42%), ENR (71.44% and 60.51%), OFX (27.02% and 29.67%), MEM (0.96% and 0.23%), and COL (0.87% and 3.27%). The AMR rate of the strains isolated from pigs and chickens showed a significant difference in most antimicrobials except AMC (*P* = 0.128), GEN (*P* = 1.267), OFX (*P* = 0.302) and MEM (*P* = 0.142). The percentages of E. coli isolated from pigs resistant to antimicrobials, such as AMP, SPT, TET, FFC, SIZ, SXT, and ENR, were significantly higher than those isolated from chickens. In contrast, there was a significantly higher prevalence of E. coli resistant to CAZ, CEF, and COL in chickens than in pigs (Fig. 2B).

Furthermore, regardless of whether E. coli was isolated from pigs or chickens, the rate of AMR in different cities was significantly different, except for the strains from pigs for tetracycline. In general, the E. coli isolated from pigs in Hangzhou, Huzhou, and Jiaxing had a higher rate of AMR than in other cities, and the strains had a lower rate of AMR in Lishui (Fig. 2C). Contrary to pigs, the E. coli isolated from chickens in Quzhou and Lishui had a higher rate of AMR than in other cities. Moreover, strains in Huzhou had a relatively higher rate of AMR against different antimicrobials (Fig. 2D).

**FIG 3:**
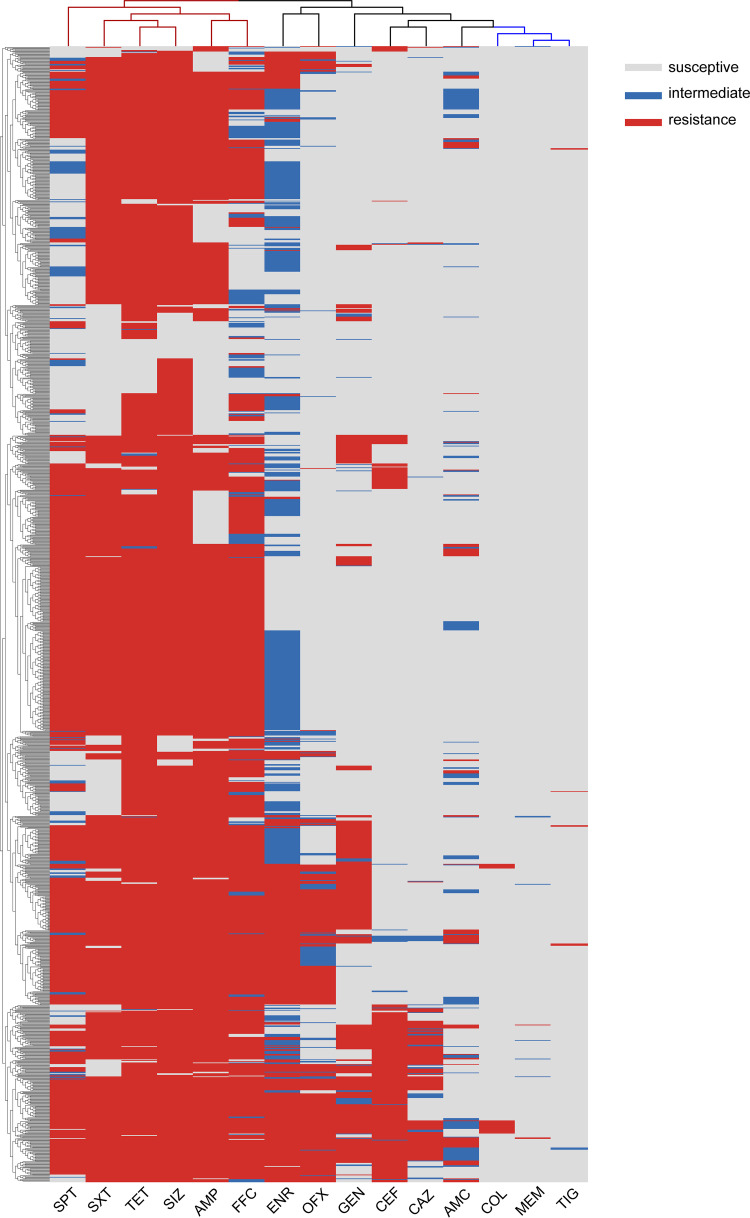
Clustering analysis of the AMR in E. coli from pigs and chickens in Zhejiang Province. The blue branch at the top of the statistical diagram shows the common coresistance antimicrobials. The red branch shows the rare AMR.

Most of the isolates (1,303, 88.68%) were MDR bacteria resistant to three or more different types of antimicrobials. Among the nine classes of antimicrobials, the isolates resistant to 5 classes of the antimicrobials were the most prevalent (445, 30.31%). A total of 215 AMR patterns were calculated, and 245 (16.69%) strains were resistant to AMP-SPT-TET-FFC-SIZ-SXT, which was the most common type (Table S1 in Supplemental File 1). Only 168 (11.44%) isolates were not MDR bacteria, and 24 of them did not show a resistant phenotype for all tested antimicrobials (Fig. 2E). The MDR rates between the isolates from pigs and chickens were significantly different. The medians were 5 and 4 in pigs and chickens, respectively (Fig. 2F). SPT, SXT, TET, SIZ, AMP, and FFC were the common MDR categories by clustering analysis (Fig. 3). The isolates from pigs have a similar AMR pattern that most strains resistant to AMP, SPT, TET, FFC, SIZ and SXT (Fig. S1 in Supplemental File 1). And the AMR pattern of isolates from chickens was more scattered than in pigs (Fig. S2 in Supplemental File 1).

### Heavy metal correlation with AMR.

The correlation analysis showed that the content of heavy metals (copper and zinc) in feed and feces was related to the AMR. The Pearson correlation coefficient showed that the copper content in the feed was significantly correlated with AMC (0.336) and TET (0.462). The copper residue in the feces was significantly correlated with AMC (0.353). The zinc content in the feed was significantly correlated with AMC (0.329) and TET (0.336). The copper and zinc residues were not correlated with any other AMRs except for AMC and TET (Table S2 in Supplemental File 1).

### Genomic associations with AMR genotypes.

**FIG 4:**
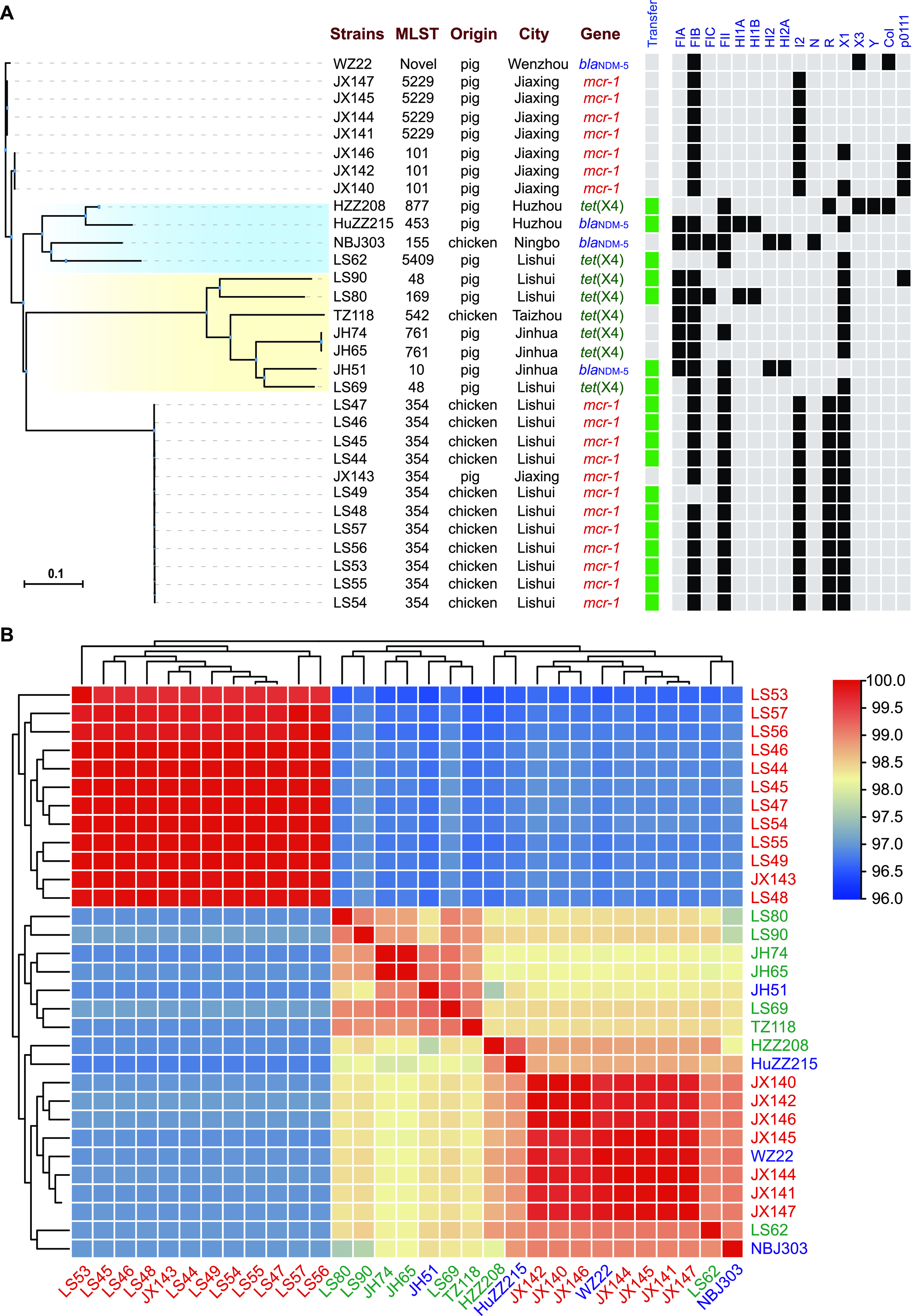
The whole-genome relatedness of 31 strains resistant to COL, TIG, and MEM from pigs and chickens in Zhejiang Province. (A) A phylogenetic tree of 31 strains was generated based on the core genome SNPs using kSNP v 3.1. The strain information is shown on the right. The capacity of the conjugative transfer and plasmid replicons are marked by black boxes. (B) The average nucleotide identity (ANI) between paired strains.

**FIG 5:**
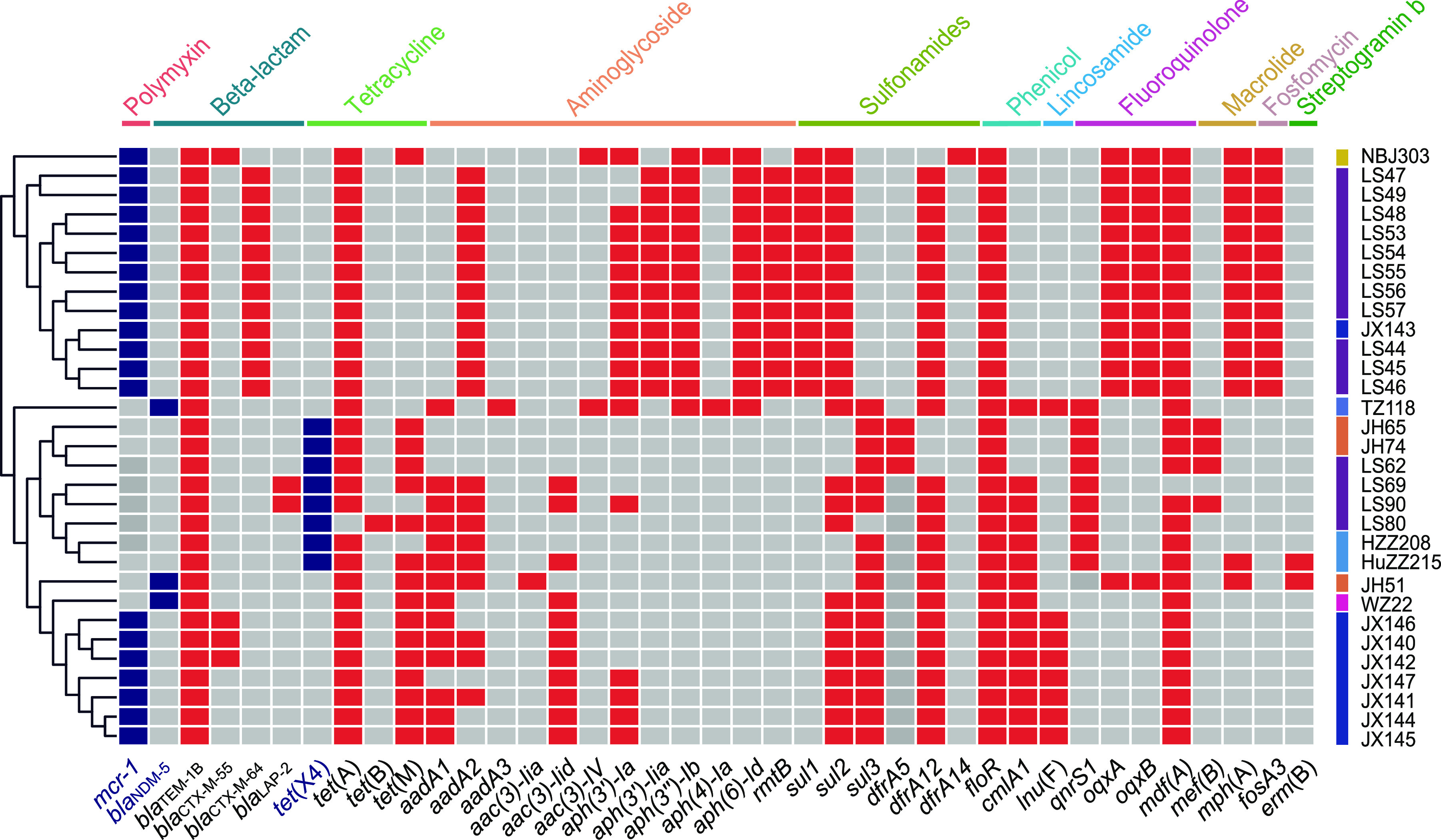
The heat map of AMR genes of the 31 strains. The *mcr-1*, *tet*(X4), and *bla*_NDM-5_ genes are marked in the blue box, and the other AMR genes are marked in the red box. The gray boxes symbolize the strain without this gene. The gene names are marked below, and the genes conferring antimicrobial resistance to the strain are marked above the diagram.

Thirty-one strains resistant to the “last-resort” antimicrobials (20 for colistin, 8 for tigecycline, and 3 for meropenem) from 1,468 strains were sequenced by the Illumina platform. Multilocus sequence typing (MLST) analysis showed that those strains belonged to 13 ST types (such as ST354, ST5229, and ST101) and one novel ST. The ST types of the strains differed among different animals and cities (Fig. 1). Phylogenetic trees of 31 strains based on SNPs showed that the *mcr-1*-positive strains had higher genetic similarity to the same city (Fig. 4A). The same ST-type strains were usually assigned to a close branch except for strains LS69 and LS90 (ST48). To verify this, the average nucleotide identity (ANI) was used to compare genome relatedness, and similar results were achieved (Fig. 4B). The AMR genotype analysis showed 38 ARGs for 11 antimicrobial classes (beta-lactam, aminoglycoside, tetracycline, polymyxin, chloramphenicol, macrolide, fosfomycin, lincosamide, fluoroquinolone, folate pathway antagonist, and streptogramin b) that were discovered in 31 strains. *bla*_TEM-1_ (31/31), *floR* (31/31), *tet*(A) (30/31), and *mdf*(A) (30/31) were the most common ARGs in 31 isolates (Fig. 5). Consistent with the phenotype, all of them contained the corresponding ARGs (*mcr-1*, *tet*(X4), or *bla*_NDM-5_).

### Plasmids and transmission mechanism.

These 31 strains contained various plasmid replicons, such as IncFIA, IncFIB, IncFIC, IncFII, IncHI1A, IncHI1B, IncHI2, IncHI2A, IncI2, IncX1, IncX3, IncY, IncN, IncR, Col, and p0111 (Fig. 4a). More than half of the strains resistant to “last-resort” antimicrobials (18/31) could transfer the ARGs to the recipient strain J53, including 11 *mcr-1*-positive strains, five *tet*(X4)-positive strains and two *bla*_NDM-5_-positive strains. The conjugation frequency was distributed from 7.5 × 10^−2^ to 9.25 × 10^−7^ (Table S3 in Supplemental File 1).

**FIG 6:**
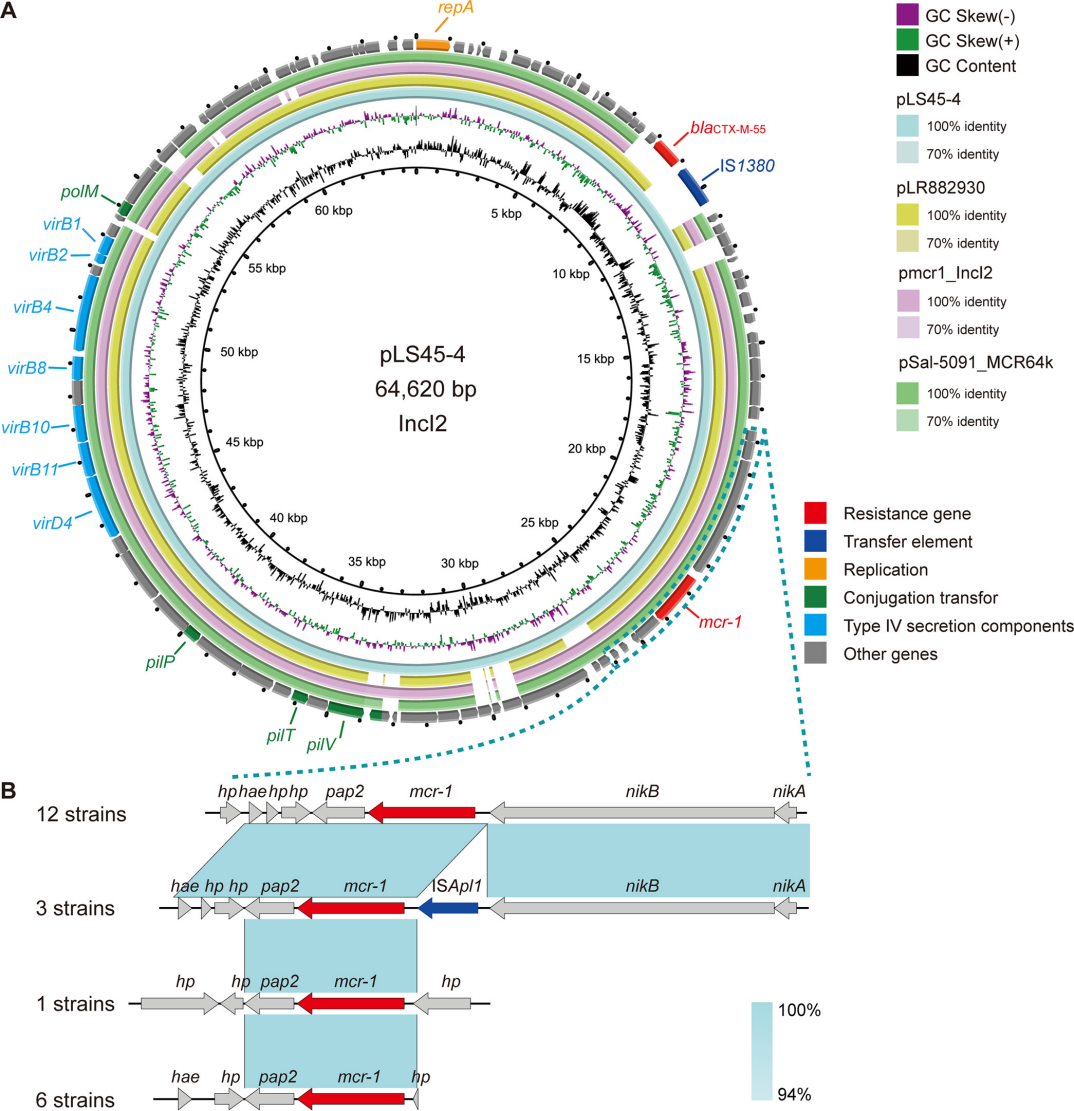
Genetic characteristics of the *mcr-1*-positive plasmid identified in this study. (A) Comparison with similar plasmids from pathogenic bacteria in humans or other animals. (B) The genetic contexts of the resistance genes are magnified under the circle graph. The arrows and triangles represent genes from different functional categories (red: resistance genes, dark blue: mobile elements, orange: replication, green: conjugation transfer, light blue: type IV secretion components, gray: other functional genes). The corresponding alignments are shown in light blue.

The LS45 strains carrying *mcr-1* and JH51 carrying *bla*_NDM-5_ transferred the ARGs to E. coli J53, which was sequenced by Oxford Nanopore Technologies for further analysis. However, the strain TZ118 carrying *tet*(X4) failed to transfer the *tet*(X4) gene to E. coli J53. The strain LS45 resistant to COL belonged to ST354 and contained four plasmids. These plasmids belonged to IncFII (pLS45-1), IncX1-R (pLS45-3), and IncI2 (pLS45-4), respectively. The *mcr-1* gene was located on the mobile plasmid pLS45-4 (IncI2, 64,620 bp). Similar plasmids carrying the *mcr-1* gene were discovered in pathogenic bacteria from humans, including E. coli (pmcr1_IncI2, KU761326), Klebsiella pneumoniae (pLR882930-1, LR882930), and Salmonella enterica (pSal-5091_MCR64k, CP045521) (Fig. 6a). The genetic context analysis showed *mcr-1* downstream of *nikA*-*nikB* (*nikA-nikB-mcr-1-pap2-hp-hp*), which was the most common genetic context in the 22 COL-resistant strains (Fig. 6b).

**FIG 7:**
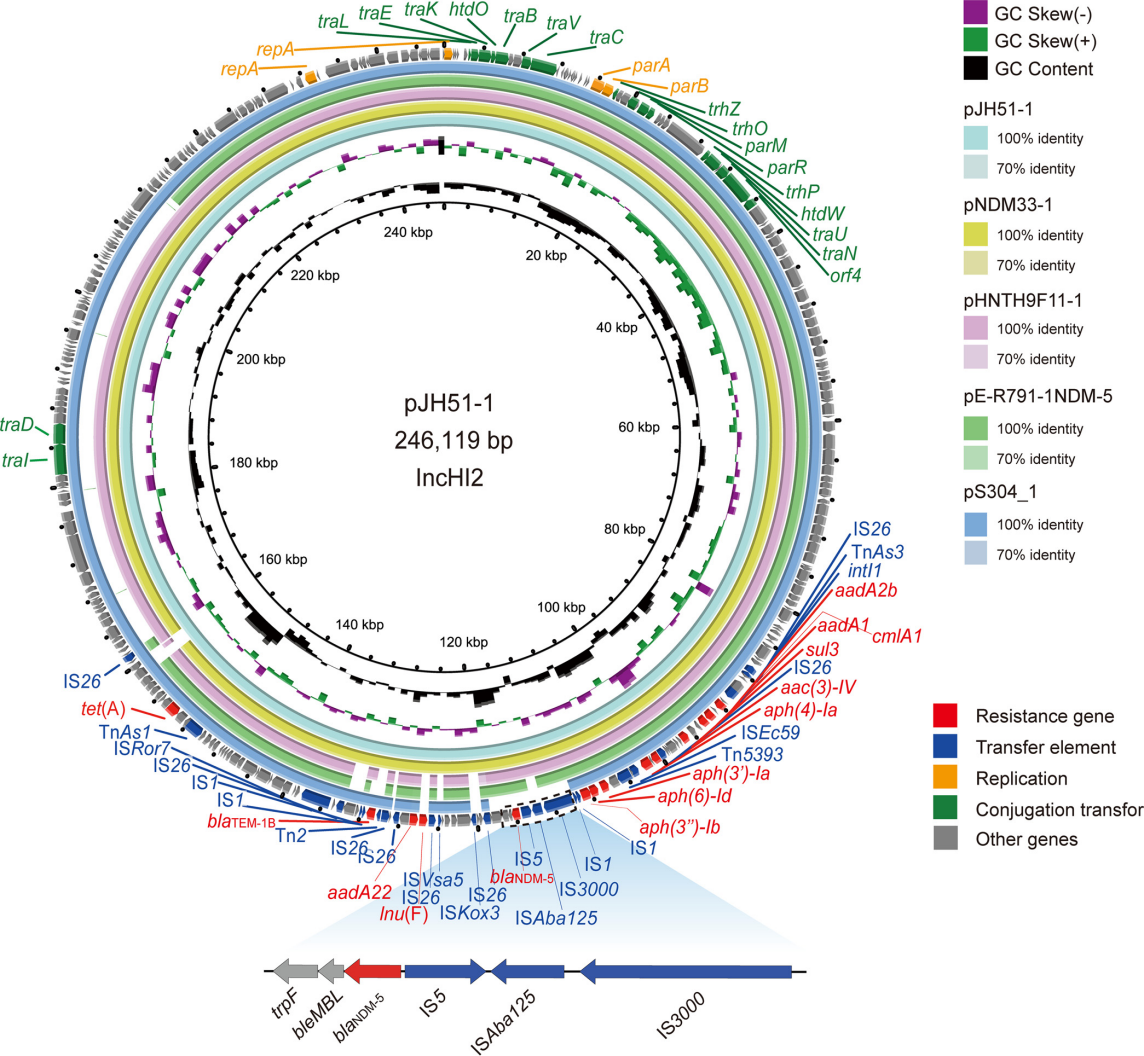
Genetic characteristics of the *bla*_NDM-5_-positive plasmid identified in this study compared with similar plasmids from pathogenic bacteria in humans or other animals. The arrows and triangles represent genes from different functional categories (red: resistance genes, dark blue: mobile elements, orange: replication, green: conjugation transfer, gray: other functional genes).

The MEM-resistant strain JH51 belonged to ST10 and contained five replicons (IncHI2, IncFII, IncFIA, IncFIB, and ColRNAI). The *bla*_NDM-5_ gene was located on the mobile plasmid pJH51-1 (IncHI2, 246,773 bp). Similar plasmids carrying the *bla*_NDM-5_ gene were only discovered in E. coli (pE-R791-1-NDM-5 (CP090263), pHNTH9F11-1 (CP054192), pNDM33-1 (MN915011)) from different origins. A similar plasmid without the *bla*_NDM-5_ gene was discovered in S. enterica (pS304_1, CP061127) from humans. The genetic contexts of *bla*_NDM-5_ were similar in E. coli in this study (IS*3000*-IS*Abal125*-IS*5*-*bla*_NDM-5_-*bleMBL*-*trpF*) (Fig. 7).

**FIG 8:**
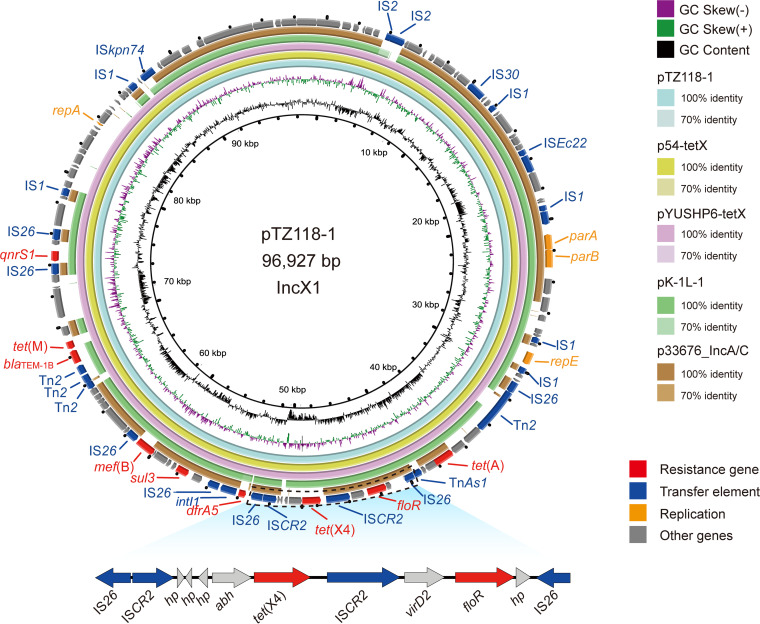
Genetic characteristics of the *tet*(X4)-positive plasmid identified in this study compared with similar plasmids from pathogenic bacteria in humans or other animals. The arrows and triangles represent genes from different functional categories (red, resistance genes; dark blue, mobile elements; orange, replication; gray, other functional genes).

Strain TZ118 resistant to TIG belonged to ST542 and involved only one plasmid (IncX1, 96,927 bp). Except for the *mdf*(A) gene, most of the ARGs in this strain were located on the plasmid, including the *tet*(X4) gene. Similar plasmids carrying the *tet*(X4) gene were discovered in E. coli from several regions (p54-tetX [CP041286], pYUSHP6-tetX [MW423609], and p1916D6-1 [CP046001]) and K. pneumoniae (pK-1L-1, CP072461). Meanwhile, a similar plasmid without the *tet*(X4) gene was identified in S. enterica (p33676_IncA/C, CP012682) from humans. The *tet*(X4) gene was located between *abh* and IS*CR2* (Fig. 8). Although pTZ118 could not transfer the *tet*(X4) gene to other strains by conjugational transfer, the mobile element (IS*CR2*) around the *tet*(X4) gene may help it transfer among bacteria horizontally.

## DISCUSSION

With the extensive use of antimicrobials in animals and humans, an increasing number of resistant bacteria have emerged in clinical patients and sick animals (2). The long-term and systematic monitoring of AMR in clinical bacteria has been used to provide an early warning index and guide the use of clinical medications, which has greatly enhanced its therapeutic effect (18, 21). Of course, there are several previous monitoring studies of AMR in animals that have been reported in China, but a limited number of samples were investigated in these studies that could not truly reflect the AMR in each region (15, 22). This is an important work from a “One Health” perspective. There has recently been a strict antimicrobial management system in China but limited systematic research on AMR in food animals. Hence, we performed large-scale AMR detection of E. coli from pig and chicken farms in all cities in Zhejiang Province, China.

In this study, 1,303 (88.68%) isolates were MDR bacteria, and only 24 (1.63%) strains were sensitive to all the antimicrobials. The AMR pattern of E. coli in this study was consistent with the expectation that the strains were highly resistant to TET, FFC, and SIZ because these antimicrobials have been widely used in the pig and poultry industry in recent years (23). This is consistent with the result of AMR phenotypes in E. coli isolates from pigs in all provinces in China, in which the isolates were highly resistant to TET (96.26%, 1,801/1,871), FFC (82.04%, 1,535/1,871) and SXT (80.38%, 1,504/1,871) (22). The AMP resistance rate in this study (78.27%, 1149/1468) was significantly higher than that reported by Peng et al. (<10%) for fewer than 50 strains of E. coli isolated from pigs in Zhejiang (22). This could be due to the small number of samples in the previous study. A higher resistance rate for AMP (92.86%, 156/168) was reported in broiler chicken fattening farms in Shandong (24). AMP has a similar resistance rate as clinical E. coli (80.5%) from hospitals (25). Furthermore, the “last-resort” AMR rates in this study were lower than the national data from pigs, especially for TIG (37.31%, 698/1,871) (22). The resistance rate of COL (1.36%) and MEM (0.75%) were lower than the national data (3.79%, 71/1,871 and 2.30%, 43/1,871, respectively) (22). The COL (0.6%), MEM (3.5%) and TIG (0.1%) resistance rates in humans were similar to those in this study (18). Similar to a previous report, the prevalence of colistin-resistant bacteria in animals decreased rapidly (from 34%, 1,153/3,396 to 5.1%, 142/2,781 in pigs, from 18.1% 474/2,614 to 5.0% 143/2,887 in chickens) with the ban of colistin as a feed additive in China in 2017 (21). Notable successes have been attained from bans on the use of colistin in animals.

The AMR pattern showed a significant difference between the strains from pigs and chickens. Similar to Peng et al. (15), the AMR rate of E. coli isolates from pigs was significantly higher than that from chickens in the vast majority of cases, such as TET (94.37% in pigs versus 89.48% in chickens), FFC (81.44% in pigs versus 40.60% in chickens), and SIZ (88.36% in pigs versus 86.42% in chickens). In contrast, the CEF, CAZ, and COL resistance rates of isolates from pigs were significantly lower than those of isolates from chickens (15). This may be caused by the choice of antimicrobials, which are different between pigs and chickens. In general, the antimicrobials used in pig breeding are more common than those used in chicken breeding (26). However, CEF and COL are more often used in poultry (15). This indicated that antimicrobial abuse could increase the AMR rate. Continuous monitoring of the AMR pattern in animal bacteria to guide the rational use of antimicrobials could be an effective method for reducing the rate of AMR.

Copper and zinc are important inorganic elements needed by animals for growth that have commonly been used as additives in animal feed. Unfortunately, an increasing number of studies have shown that heavy metal residues are associated with AMR in bacteria (27). This study also discovered correlations of copper and zinc with TET and AMC resistance. Copper can accelerate the conjugative transfer of ARGs between bacteria, and heavy metal resistance genes have always been discovered in the same plasmid as ARGs (28, 29). Much more attention should be directed toward controlling the use of antimicrobials, along with controlling heavy metals as additives in feed.

It should be noted that the COL, TIG, and MEM resistant strains were identified from pigs and chickens in several cities. However, the rate of these resistant strains was lower than that in other regions in China (15, 22). These antimicrobials, especially carbapenems, have been prohibited as animal feed additives and as treatments for animals. The *mcr-1* gene exists because of the widespread use of COL in feed among animals, and its prevalence has significantly decreased since the ban (30). SNP and MLST analyses showed that the *mcr-1*-positive strains were more closely related than *tet*(X4) and *bla*_NDM-5_. This means that the *mcr-1*-positive strains exhibited clonal spread and that the *tet*(X4)- and *bla*_NDM-5_-positive strains were sparsely distributed. The *tet*(X4) gene may be associated with long-term TET use in animals. The *bla*_NDM-5_ gene was discovered in a small number of bacteria that might be associated with human activities. Unfortunately, this study did not collect feces samples from farmworkers, which could help us understand these ARGs and provide information about their transfer between animals and humans. There is no strain harboring these three genes at present.

Plasmid-mediated horizontal gene transfer could dramatically increase the prevalence of ARGs. *mcr-1*, *tet*(X4), and *bla*_NDM-5_ conferred resistance to the COL, TIG, and MEM strains in this study. The conjugational transfer tests showed that more than half of these strains could transmit the ARGs horizontally by plasmids. In this study, most *mcr-1* was located on the IncI2 plasmid, and IncI2 is one of the most common types of plasmids associated with *mcr-1* (30). The *mcr-1*-positive IncI2 plasmid has also been identified in clinical isolates (31). Moreover, IncHI2 was also one of the common plasmids carrying the *mcr-1* gene in a previous study (32). This study found that the *bla*_NDM-5_-positive plasmid pJH51 belongs to IncHI2, which may increase the risk of cotransfer of the *mcr-1* gene.

WGS showed the *tet*(X4) located on the IncX1 plasmid in strain TZ118, and IncX1 is usually a mobile plasmid type (33). Contrary to expectations, pTZ118 harboring *tet*(X4) could not be transferred to recipient strains by conjugational transfer. Further analysis of the sequence showed that pTZ118 lacked the essential elements (mob/tra-locus, type IV secretion system, and genes involved in DNA transfer and post conjugational replication) for the conjugational system (34). This may reduce the prevalence of *tet*(X4) in bacteria. However, the insertion element IS*CR2* around *tet*(X4) could generate a circular intermediate to transfer the ARG independently of plasmids (35). The prevalence and transmission mechanism of the *tet*(X4) gene should be given more attention.

The same types of plasmids carrying the ARGs were discovered in E. coli from different regions or origins. They had a higher risk of transmission between E. coli in animals, humans, and the environment. In addition, similar plasmids were discovered in K. pneumoniae and S. enterica, indicating that these important ARGs may be transferred to the pathogens. We need to be alert to the potential risk that these pathogens can capture ARGs from E. coli.

In summary, our results demonstrated that E. coli strains were generally resistant to TET, SIZ, and FFC under a situation of widespread use of antimicrobials. Although the strains from different animals and cities exhibited different AMR patterns, most of them were multidrug resistant. Most of the *mcr-1*, *tet*(X4), and *bla*_NDM-5_ genes could be transferred between the bacteria on the plasmids discovered in this study. Moreover, the ban of colistin as a feed additive in animals effectively reduced the prevalence of *mcr-1* genes. This is an efficient way to reduce the AMR rates based on the monitoring of the strain AMR patterns and regular adjustment. It is important to reduce the MDR bacterial threat to human health through the food chain.

## MATERIALS AND METHODS

### Sample collection.

This study was conducted in all cities (Hangzhou, Ningbo, Huzhou, Jinhua, Shaoxing, Wenzhou, Jiaxing, Taizhou, Quzhou, Lishui, and Zhoushan) of Zhejiang Province, China. These regions included hills, plains, and islands. A total of 1,505 feces and anal swab samples were collected randomly from the healthy pigs (1,060) and chickens (445) on farms in every city between January 2020 and December 2020 (Table 1). The samples were kept in a cryogenic incubator (4 to 8°C) and sent to the laboratory as soon as possible.

### Isolation and identification of E. coli.

Each sample was placed in a separate 10 mL asepsis pipe with 5 mL of buffered peptone water (BPW) and shaken hard. Then, 500 μL of the mixture was placed in a new asepsis pipe with 5 mL BPW and incubated at 37°C for 12 h. A 10 μL aliquot from each sample was inoculated on MacConkey agar plates and incubated at 37°C for 16 h. The putative E. coli single colonies were inoculated on eosin methylene blue media and LB agar and then confirmed by MALDI-TOFMS.

### Antimicrobial susceptibility testing.

All of the E. coli isolates were tested for antimicrobial susceptibility using 15 antimicrobials (ampicillin (AMP), amoxicillin/clavulanate (AMC), gentamicin (GEN), spectinomycin (SPT), florfenicol (FFC), sulfisoxazole (SIZ), sulfamethoxazole/trimethoprim (SXT), ceftiofur (CEF), ceftazidime (CAZ), enrofloxacin (ENR), ofloxacin (OFX), tetracycline (TET), tigecycline (TIG), colistin (COL) and meropenem (MEM)) using the broth microdilution method according to the Clinical and Laboratory Standards Institute guidelines (CLSI, 2019). The colistin-, tigecycline- and meropenem-resistant strains were verified by inoculation on an MH agar plate containing the corresponding antimicrobials.

### Conjugational transfer.

Colistin-, meropenem-, or tigecycline-resistant E. coli containing the *mcr-1*, *tet*(X4), or *bla*_NDM-5_ genes were used as the donor strain, and the E. coli strain J53 (sodium azide resistance) was used as the recipient strain. The donor and recipient strains were cultured to the logarithmic growth stage, and then they were cultured together overnight after adjusting their concentration. The mixture was diluted with a PBS gradient and then inoculated onto LB plates containing sodium azide (100 mg/L) and appropriate antimicrobials (COL [2 mg/L], TIG [2 mg/L], or MEM [2 mg/L]). The precise method of the conjugational transfer experiment was conducted as described in a previous study (36).

### Whole-genome sequencing and analysis (WGS).

The strains were incubated in 5 mL of LB broth at 37°C shaking at 180 rpm overnight and then transferred to a fresh culture medium at a 1:100 ratio and allowed to grow for another 2 to 3 h. The cells were collected by centrifugation at 10,000 rpm/min, and the genome was extracted using a DNA Extraction kit (Genray, Shanghai). Then, 31 strains resistant to COL, MEM, or TIG were sequenced by Illumina sequencing (Illumina HiSeq-PE150) and assembled by CLC Genomics Workbench 12.0. The LS45, JH51, and TZ118 strains were further sequenced by the Oxford Nanopore GridION platform and assembled by Unicycler v0.4.4. The sequences were annotated by RAST (37). The ARGs, MLST, and Inc. type of plasmids were analyzed by the Center for Genomic Epidemiology (CGE, http://www.genomicepidemiology.org/). The heatmap was generated by TBtools v1.0 (38). The results of comparative genome analysis were visualized using BRIG v0.95 and EasyFig v2.2.3 (39, 40). The phylogenetic tree based on the core genome SNPs was generated by kSNP3.0 (41).

### Determination of heavy metal content.

A total of 33 fecal and 35 feed samples were collected from livestock and poultry farms in 5 cities (Jinhua, Wenzhou, Taizhou, Jiaxing, and Lishui). The copper and zinc contents in the samples were measured by inductively coupled plasma-mass spectrometry (ICP-MS) (Agilent Technologies, Santa Clara, CA, USA) as described in a previous study (42). In short, 0.5 g samples were placed in a Teflon digestion vessel, and nitric acid was added. After heating, perchloric acid and hydrochloric acid were added in turn. Then, the mixture was diluted with distilled water to 25 mL for testing.

### Statistical analysis.

The data for several factors affecting the AMR rates in E. coli were analyzed with chi-square tests by SPSS v19.0 (IBM Corporation, Somers, NY). A probability (*P*) value <0.05 was considered statistically significant. The correlation between the content of heavy metals and the AMR in E. coli was analyzed by SPSS using Pearson’s correlation.

### Data availability.

The whole-genome sequences (WGSs) of the E. coli isolates in this study have been deposited in GenBank under accession code PRJNA822011. The GenBank accession numbers of strains LS45, TZ118, and JH51 were CP095448 to CP095453, CP095446 to CP095447, and CP095454 to CP095460, respectively.
