# Supplementary material for: Large-Scale Studies on Antimicrobial Resistance and Molecular Characterization of Escherichia coli from Food Animals in Developed Areas of Eastern China
Source: Microbiol Spectr. 2022 Aug 11;10(4):e02015-22. doi: 10.1128/spectrum.02015-22 (PMC9430128; doi:10.1128/spectrum.02015-22)
Supplement: Supplemental file 1 — Supplemental material. Download spectrum.02015-22-s0001.pdf, PDF file, 1.3 MB [file spectrum.02015-22-s0001.pdf]

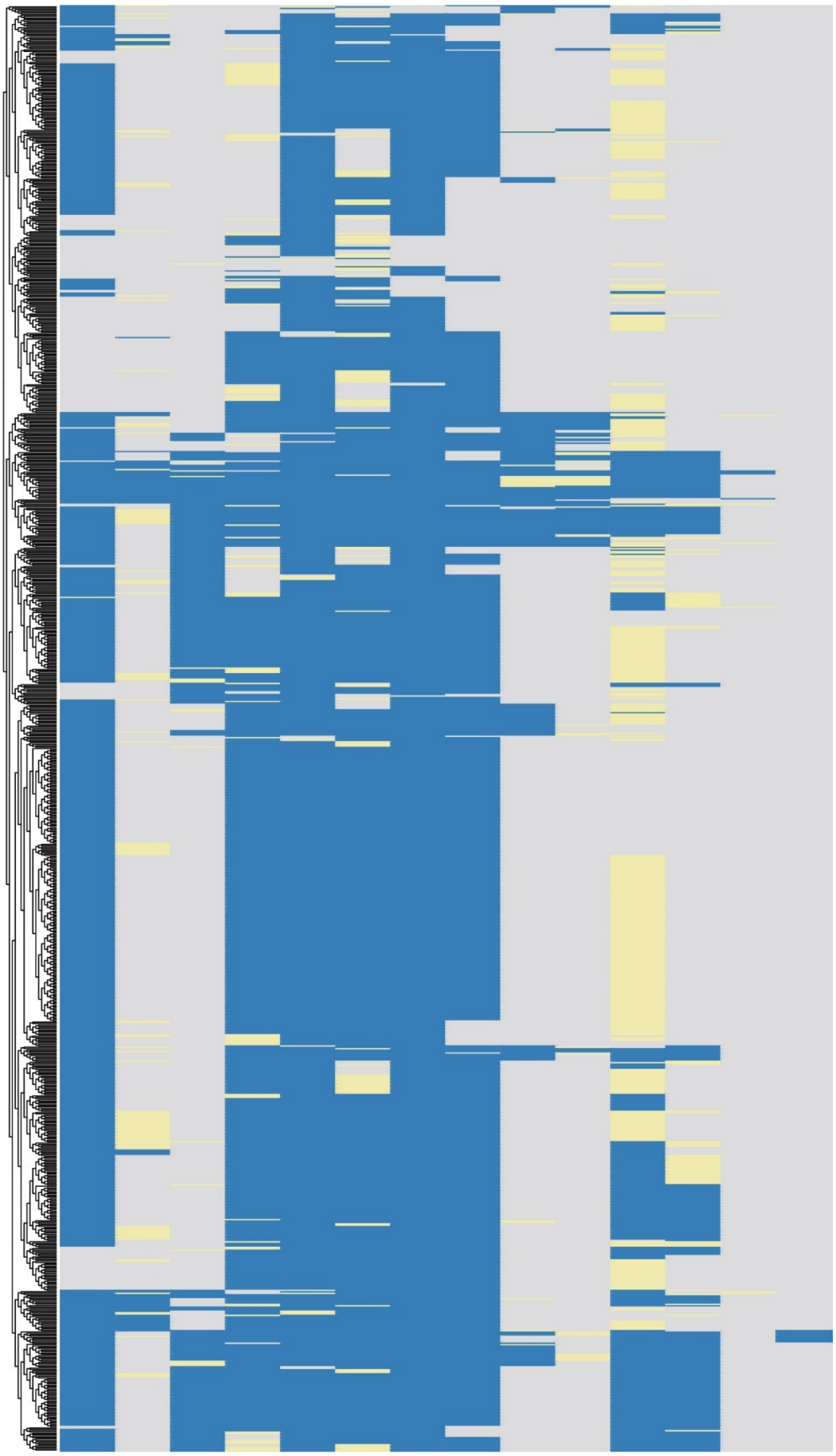

Fig. S1 The AMR patterns of the isolates from pigs. The blue represents the resistant pattern, and the pale-yellow boxes are intermediate.

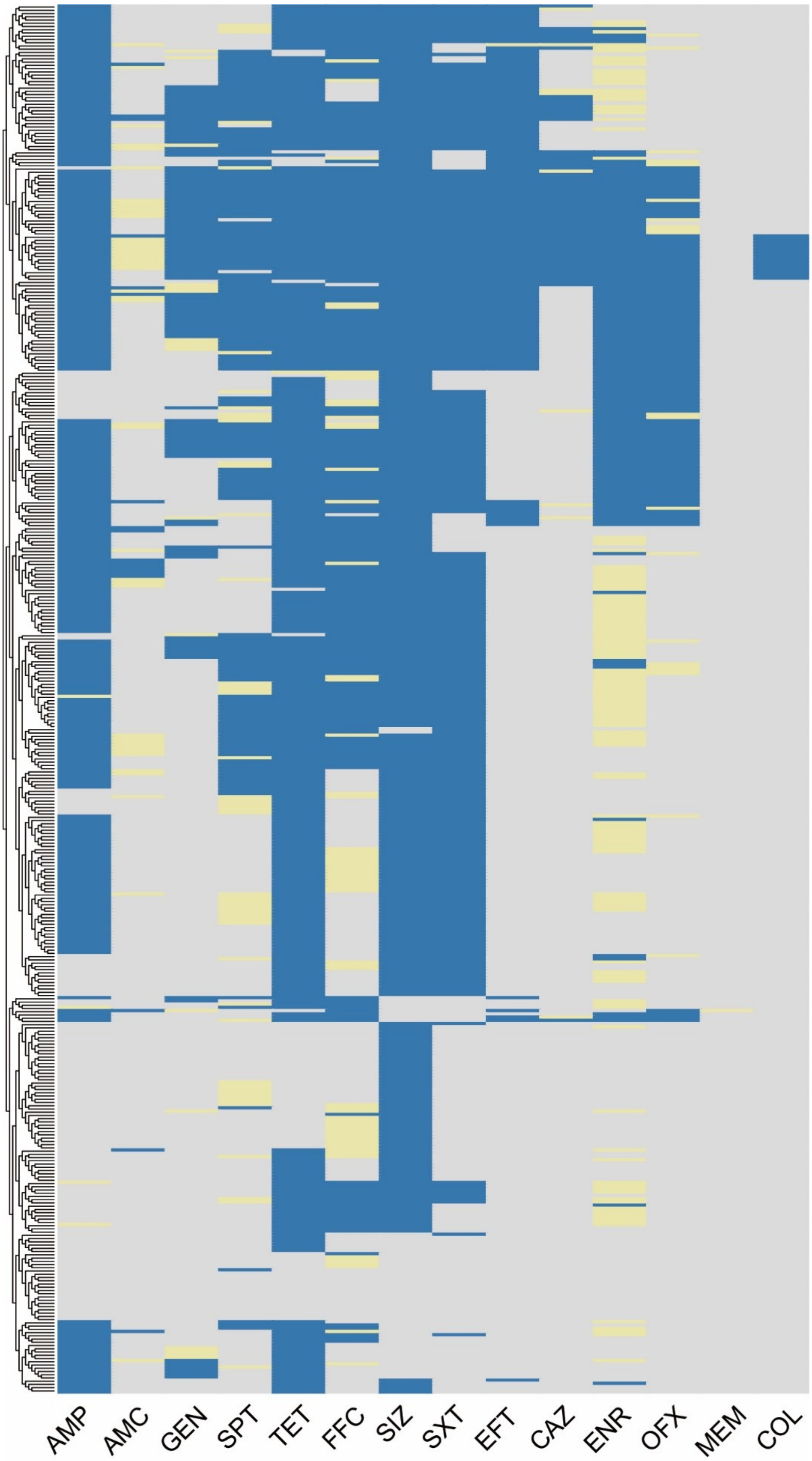

Fig. S2 The resistance pattern of the isolates from chickens. The blue boxes represent the resistant pattern, and the pale-yellow boxes are intermediate.

Table S1 AMR patterns of 1,468 *E. coli* strains.

Antibiotic classes	Antibiotics	AMR pattern	Isolate number	Percentage (%)
0	0	0	24	1.64
1	1	SIZ	41	2.8
1	1	TET	11	0.75
1	1	SPT	2	0.14
1	1	FFC	2	0.14
1	2	SIZ-SXT	1	0.07
2	3	TET-SIZ-SXT	29	1.98
2	2	TET-SIZ	21	1.44
2	2	SPT-TET	7	0.48
2	2	AMP-TET	6	0.41
2	3	SPT-SIZ-SXT	3	0.21
2	2	TET-FFC	3	0.21
2	2	AMP-CEF	3	0.21
2	3	SIZ-ENR-OFX	2	0.14
2	2	FFC-SIZ	2	0.14
2	2	SPT-SIZ	2	0.14
2	3	AMP-SIZ-SXT	2	0.14
2	2	TET-SXT	2	0.14
2	3	GEN-SPT-TET	1	0.07
2	3	AMP-AMC-TET	1	0.07
3	4	AMP-TET-SIZ-SXT	72	4.91
3	3	TET-FFC-SIZ	25	1.71
3	4	SPT-TET-SIZ-SXT	19	1.3
3	4	TET-FFC-SIZ-SXT	15	1.03
3	3	AMP-TET-SIZ	10	0.69
3	3	AMP-TET-FFC	6	0.41
3	3	AMP-GEN-TET	6	0.41
3	4	TET-SIZ-ENR-OFX	5	0.35
3	5	AMP-AMC-TET-SIZ-SXT	3	0.21
3	3	SPT-TET-SIZ	3	0.21
3	4	TET-SIZ-SXT-ENR	3	0.21
3	4	AMP-FFC-SIZ-SXT	2	0.14
3	3	SPT-TET-SXT	2	0.14
3	3	SPT-TET-FFC	2	0.14
3	5	GEN-SPT-TET-SIZ-SXT	2	0.14
3	4	SPT-FFC-SIZ-SXT	2	0.14
3	5	TET-SIZ-SXT-ENR-OFX	2	0.14
3	3	AMP-SPT-TET	2	0.14
3	4	AMP-SXT-CEF-CAZ	1	0.07
3	3	AMP-TET-CEF	1	0.07
3	4	GEN-SPT-TET-SIZ	1	0.07

3	4	AMP-FFC-CEF-CAZ	1	0.07
3	4	AMP-SIZ-CEF-CAZ	1	0.07
3	3	GEN-TET-FFC	1	0.07
3	3	AMC-TET-SIZ	1	0.07
3	4	AMP-AMC-TET-FFC	1	0.07
4	5	AMP-TET-FFC-SIZ-SXT	69	4.71
4	5	SPT-TET-FFC-SIZ-SXT	46	3.14
4	4	AMP-TET-FFC-SIZ	33	2.25
4	5	AMP-SPT-TET-SIZ-SXT	32	2.18
4	5	AMP-GEN-TET-SIZ-SXT	8	0.55
4	6	AMP-AMC-TET-FFC-SIZ-SXT	7	0.48
4	4	AMP-SPT-TET-FFC	4	0.28
4	5	AMP-SPT-FFC-SIZ-SXT	4	0.28
4	5	AMP-AMC-TET-FFC-SIZ	4	0.28
4	6	GEN-SPT-TET-FFC-SIZ-SXT	4	0.28
4	5	AMP-GEN-FFC-SIZ-SXT	4	0.28
4	4	AMP-GEN-TET-SIZ	3	0.21
4	6	AMP-GEN-SPT-TET-SIZ-SXT	3	0.21
4	6	SPT-TET-SIZ-SXT-ENR-OFX	3	0.21
4	4	TET-FFC-SIZ-ENR	3	0.21
4	6	TET-FFC-SIZ-SXT-ENR-OFX	3	0.21
4	6	AMP-TET-SIZ-SXT-ENR-OFX	3	0.21
4	7	GEN-SPT-TET-SIZ-SXT-ENR-OFX	2	0.14
4	5	AMP-TET-SIZ-SXT-CAZ	2	0.14
4	6	AMP-TET-FFC-SIZ-SXT-TIG	2	0.14
4	4	AMP-TET-FFC-SXT	2	0.14
4	4	GEN-TET-FFC-SIZ	2	0.14
4	5	GEN-TET-FFC-SIZ-SXT	2	0.14
4	4	SPT-TET-FFC-SIZ	2	0.14
4	5	TET-FFC-SIZ-SXT-ENR	1	0.07
4	5	AMP-TET-SIZ-SXT-CEF	1	0.07
4	5	AMP-FFC-SIZ-SXT-CEF	1	0.07
4	4	AMP-TET-SIZ-ENR	1	0.07
4	6	GEN-TET-SIZ-SXT-ENR-OFX	1	0.07
4	4	AMP-TET-SIZ-CEF	1	0.07
4	5	AMP-TET-SIZ-SXT-ENR	1	0.07
4	5	AMP-TET-FFC-SIZ-TIG	1	0.07
4	4	SPT-TET-FFC-ENR	1	0.07
4	5	AMP-AMC-FFC-CEF-OFX	1	0.07
4	5	SPT-TET-SIZ-CEF-CAZ	1	0.07
4	4	AMP-SPT-FFC-SIZ	1	0.07
4	4	SPT-TET-FFC-SXT	1	0.07
4	6	AMP-AMC-SPT-FFC-SIZ-SXT	1	0.07
4	7	AMP-AMC-TET-SIZ-SXT-ENR-OFX	1	0.07

5	6	AMP-SPT-TET-FFC-SIZ-SXT	245	16.69
5	7	AMP-GEN-SPT-TET-FFC-SIZ-SXT	49	3.34
5	6	AMP-GEN-TET-FFC-SIZ-SXT	13	0.89
5	6	AMP-TET-FFC-SIZ-SXT-CEF	11	0.75
5	7	AMP-AMC-SPT-TET-FFC-SIZ-SXT	10	0.69
5	5	AMP-SPT-TET-FFC-SIZ	10	0.69
5	7	AMP-TET-FFC-SIZ-SXT-ENR-OFX	7	0.48
5	6	AMP-TET-FFC-SIZ-SXT-ENR	7	0.48
5	6	AMP-SPT-TET-SIZ-SXT-CEF	6	0.41
5	5	AMP-GEN-TET-FFC-SIZ	6	0.41
5	7	AMP-GEN-TET-SIZ-SXT-ENR-OFX	5	0.35
5	6	AMP-SPT-TET-SIZ-SXT-ENR	5	0.35
5	8	AMP-GEN-SPT-TET-SIZ-SXT-ENR-OFX	5	0.35
5	5	AMP-TET-FFC-SIZ-CEF	4	0.28
5	7	SPT-TET-FFC-SIZ-SXT-ENR-OFX	4	0.28
5	7	AMP-TET-FFC-SIZ-SXT-CEF-CAZ	3	0.21
5	5	AMP-SPT-TET-FFC-SXT	3	0.21
5	7	AMP-GEN-SPT-TET-SIZ-SXT-CEF	3	0.21
5	8	AMP-AMC-GEN-SPT-TET-FFC-SIZ-SXT	3	0.21
5	6	SPT-TET-FFC-SIZ-SXT-ENR	3	0.21
5	7	AMP-SPT-TET-SIZ-SXT-ENR-OFX	3	0.21
5	5	AMP-GEN-TET-SIZ-ENR	2	0.14
5	8	AMP-GEN-SPT-TET-SIZ-SXT-CEF-CAZ	2	0.14
5	8	AMP-GEN-SPT-FFC-SIZ-SXT-ENR-OFX	2	0.14
5	7	GEN-SPT-TET-FFC-SIZ-ENR-OFX	2	0.14
5	6	AMP-SPT-SIZ-CEF-CAZ-ENR	1	0.07
5	7	AMP-GEN-SPT-SIZ-CEF-CAZ-ENR	1	0.07
5	6	AMP-GEN-TET-SIZ-SXT-ENR	1	0.07
5	6	AMP-TET-FFC-SIZ-ENR-OFX	1	0.07
5	6	GEN-TET-FFC-SIZ-CEF-CAZ	1	0.07
5	7	AMP-AMC-GEN-FFC-SIZ-SXT-ENR	1	0.07
5	6	AMP-GEN-FFC-SIZ-SXT-ENR	1	0.07
5	7	GEN-TET-FFC-SIZ-SXT-ENR-OFX	1	0.07
5	8	AMP-GEN-SPT-TET-FFC-SIZ-SXT-TIG	1	0.07
5	6	AMC-SPT-TET-FFC-SIZ-SXT	1	0.07
5	6	AMP-GEN-SPT-TET-FFC-SIZ	1	0.07
5	6	AMP-TET-FFC-SIZ-CEF-CAZ	1	0.07
5	7	GEN-SPT-TET-FFC-SIZ-SXT-ENR	1	0.07
5	7	AMP-AMC-TET-FFC-SIZ-SXT-ENR	1	0.07
5	6	AMP-TET-FFC-SIZ-SXT-CAZ	1	0.07
5	5	AMP-TET-FFC-ENR-OFX	1	0.07
5	6	AMP-TET-FFC-CEF-ENR-OFX	1	0.07
5	7	AMP-TET-FFC-CEF-CAZ-ENR-OFX	1	0.07
5	6	AMP-GEN-SPT-TET-FFC-CEF	1	0.07

5	7	AMP-GEN-FFC-SIZ-SXT-CEF-CAZ	1	0.07
5	5	AMP-TET-FFC-SIZ-ENR	1	0.07
5	5	AMP-SPT-FFC-SIZ-CEF	1	0.07
5	6	AMP-SPT-FFC-SIZ-SXT-CEF	1	0.07
5	5	AMP-SPT-TET-FFC-ENR	1	0.07
5	5	AMP-SPT-TET-SIZ-CEF	1	0.07
5	8	AMP-AMC-TET-SIZ-SXT-CEF-ENR-OFX	1	0.07
5	6	AMP-TET-SIZ-CEF-ENR-OFX	1	0.07
5	5	AMP-TET-FFC-SIZ-CAZ	1	0.07
6	9	AMP-GEN-SPT-TET-FFC-SIZ-SXT-ENR-OFX	49	3.34
6	8	AMP-SPT-TET-FFC-SIZ-SXT-ENR-OFX	44	3
6	7	AMP-SPT-TET-FFC-SIZ-SXT-ENR	38	2.59
6	7	AMP-SPT-TET-FFC-SIZ-SXT-CEF	19	1.3
6	9	AMP-GEN-SPT-TET-FFC-SIZ-SXT-CEF-CAZ	10	0.69
6	8	AMP-GEN-SPT-TET-FFC-SIZ-SXT-CEF	10	0.69
6	10	AMP-AMC-GEN-SPT-TET-FFC-SIZ-SXT-ENR-OFX	9	0.62
6	8	AMP-GEN-SPT-TET-FFC-SIZ-SXT-ENR	8	0.55
6	8	AMP-SPT-TET-FFC-SIZ-SXT-CEF-CAZ	6	0.41
6	8	AMP-GEN-TET-FFC-SIZ-SXT-CEF-CAZ	5	0.35
6	9	AMP-AMC-SPT-TET-FFC-SIZ-SXT-ENR-OFX	5	0.35
6	8	AMP-GEN-TET-FFC-SIZ-SXT-ENR-OFX	5	0.35
6	7	AMP-GEN-TET-FFC-SIZ-SXT-ENR	4	0.28
6	10	AMP-AMC-GEN-SPT-TET-FFC-SIZ-SXT-CEF-CAZ	4	0.28
6	8	AMP-AMC-SPT-TET-FFC-SIZ-SXT-ENR	4	0.28
6	8	AMP-TET-FFC-SIZ-SXT-CEF-CAZ-ENR	4	0.28
6	7	AMP-GEN-TET-FFC-SIZ-ENR-OFX	4	0.28
6	7	AMP-GEN-TET-FFC-SIZ-SXT-CEF	3	0.21
6	6	AMP-SPT-TET-FFC-SIZ-CEF	3	0.21
6	8	AMP-TET-FFC-SIZ-SXT-CEF-ENR-OFX	3	0.21
6	9	AMP-GEN-SPT-TET-SIZ-SXT-CEF-ENR-OFX	2	0.14
6	9	AMP-AMC-SPT-TET-FFC-SIZ-SXT-CEF-CAZ	2	0.14
6	9	AMP-SPT-TET-FFC-SIZ-SXT-ENR-OFX-TIG	2	0.14
6	6	AMP-SPT-TET-FFC-SIZ-ENR	2	0.14
6	9	AMP-SPT-TET-SIZ-SXT-CEF-CAZ-ENR-OFX	2	0.14
6	7	AMP-SPT-FFC-SIZ-CEF-CAZ-ENR	1	0.07
6	8	AMP-GEN-SPT-TET-FFC-SIZ-ENR-OFX	1	0.07
6	11	AMP-AMC-GEN-SPT-TET-SIZ-SXT-CEF-CAZ-ENR-OFX	1	0.07
6	7	AMP-SPT-FFC-SIZ-SXT-CEF-OFX	1	0.07
6	9	AMP-GEN-SPT-TET-FFC-SIZ-SXT-ENR-TIG	1	0.07
6	9	AMP-AMC-GEN-SPT-TET-FFC-SIZ-SXT-CEF	1	0.07
6	9	GEN-SPT-TET-FFC-SIZ-SXT-CEF-CAZ-ENR	1	0.07

6	8	AMC-TET-FFC-SIZ-SXT-CEF-ENR-OFX	1	0.07
6	8	AMP-AMC-GEN-TET-FFC-SIZ-SXT-ENR	1	0.07
6	8	AMP-AMC-SPT-TET-FFC-SIZ-SXT-CEF	1	0.07
6	8	AMP-SPT-TET-FFC-SIZ-SXT-ENR-TIG	1	0.07
6	9	AMP-SPT-FFC-SIZ-SXT-CEF-CAZ-ENR-OFX	1	0.07
6	8	AMP-GEN-SPT-TET-SIZ-CEF-CAZ-ENR	1	0.07
6	7	AMP-TET-FFC-SIZ-SXT-CEF-ENR	1	0.07
6	7	AMP-TET-FFC-SIZ-CEF-ENR-OFX	1	0.07
6	7	AMP-GEN-SPT-TET-FFC-SIZ-ENR	1	0.07
6	7	AMP-SPT-TET-FFC-SIZ-ENR-OFX	1	0.07
6	7	AMP-SPT-TET-FFC-SIZ-CEF-CAZ	1	0.07
6	7	AMP-SPT-TET-FFC-SIZ-SXT-OFX	1	0.07
6	9	AMP-AMC-GEN-SPT-TET-FFC-SIZ-SXT-ENR	1	0.07
6	8	GEN-TET-FFC-SIZ-CEF-CAZ-ENR-OFX	1	0.07
7	11	AMP-GEN-SPT-TET-FFC-SIZ-SXT-CEF-CAZ-ENR-OFX	30	2.05
7	10	AMP-GEN-SPT-TET-FFC-SIZ-SXT-CEF-ENR-OFX	24	1.64
7	9	AMP-SPT-TET-FFC-SIZ-SXT-CEF-ENR-OFX	19	1.3
7	12	AMP-AMC-GEN-SPT-TET-FFC-SIZ-SXT-CEF-CAZ-ENR-OFX	9	0.62
7	10	AMP-GEN-SPT-TET-FFC-SIZ-SXT-ENR-OFX-COL	5	0.35
7	10	AMP-GEN-SPT-TET-FFC-SIZ-SXT-CEF-CAZ-ENR	5	0.35
7	10	AMP-GEN-SPT-TET-FFC-SIZ-CEF-CAZ-ENR-OFX	4	0.28
7	11	AMP-GEN-SPT-TET-FFC-SIZ-SXT-CEF-ENR-OFX-COL	3	0.21
7	10	AMP-AMC-SPT-TET-FFC-SIZ-SXT-CEF-ENR-OFX	3	0.21
7	11	AMP-AMC-GEN-SPT-TET-FFC-SIZ-SXT-CEF-ENR-OFX	3	0.21
7	8	AMP-GEN-TET-FFC-SIZ-CEF-ENR-OFX	2	0.14
7	9	AMP-GEN-SPT-TET-FFC-SIZ-CEF-ENR-OFX	2	0.14
7	9	AMP-SPT-TET-FFC-SIZ-SXT-CEF-CAZ-ENR	2	0.14
7	10	AMP-SPT-TET-FFC-SIZ-SXT-CEF-CAZ-ENR-OFX	2	0.14
7	9	AMP-GEN-SPT-TET-FFC-SIZ-SXT-CEF-ENR	2	0.14
7	9	AMP-GEN-TET-FFC-SIZ-SXT-CEF-CAZ-ENR	1	0.07
7	13	AMP-AMC-GEN-SPT-TET-FFC-SIZ-SXT-CEF-CAZ-ENR-OFX-MEM	1	0.07
7	10	AMP-GEN-TET-FFC-SIZ-SXT-CEF-CAZ-ENR-OFX	1	0.07

7	11	AMP-AMC-GEN-SPT-TET-FFC-SIZ-SXT-CEF-CAZ-MEM	1	0.07
7	8	AMP-SPT-TET-FFC-SIZ-SXT-CEF-OFX	1	0.07
7	13	AMP-AMC-GEN-SPT-TET-FFC-SIZ-SXT-CEF-CAZ-ENR-OFX-COL	1	0.07
7	10	AMP-AMC-SPT-TET-FFC-SIZ-SXT-CEF-CAZ-ENR	1	0.07
7	10	AMP-GEN-SPT-TET-FFC-SIZ-SXT-CAZ-ENR-OFX	1	0.07
7	10	AMP-AMC-GEN-TET-FFC-SIZ-CEF-CAZ-ENR-OFX	1	0.07
7	10	AMP-AMC-GEN-SPT-TET-FFC-SIZ-SXT-ENR-COL	1	0.07
7	8	AMP-SPT-TET-FFC-SIZ-CEF-ENR-OFX	1	0.07
7	8	AMP-SPT-TET-FFC-SIZ-SXT-CEF-ENR	1	0.07
7	9	AMP-GEN-TET-FFC-SIZ-SXT-CEF-ENR-OFX	1	0.07
7	11	AMP-AMC-GEN-SPT-TET-FFC-SIZ-SXT-CAZ-ENR-OFX	1	0.07
7	11	AMP-AMC-TET-FFC-SIZ-SXT-CEF-CAZ-ENR-OFX-MEM	1	0.07
8	12	AMP-GEN-SPT-TET-FFC-SIZ-SXT-CEF-CAZ-ENR-OFX-COL	12	0.82
8	11	AMP-GEN-TET-FFC-SIZ-SXT-CEF-CAZ-ENR-OFX-COL	1	0.07

Table S2 The Pearson correlation coefficients between the content of heavy metals in feces or feed and the antimicrobial resistance of *E. coli*.

category	Pearson correlation	Copper		Zinc	
		in feces	in feed	in feces	in feed
AMP	coefficient	0.302	0.312	0.109	0.207
	p values	0.066	0.057	0.515	0.213
AMC	coefficient	0.353*	0.336*	0.087	0.329*
	p values	0.03	0.039	0.603	0.044
GEN	coefficient	0.062	0.027	-0.056	-0.024
	p values	0.713	0.871	0.739	0.885
SPT	coefficient	0.131	0.133	-0.029	0.021
	p values	0.434	0.426	0.861	0.9
TET	coefficient	0.274	0.462**	0.156	0.336*
	p values	0.097	0.003	0.351	0.039
FFC	coefficient	0.258	0.196	0.114	0.18
	p values	0.118	0.239	0.495	0.279
SIZ	coefficient	0.237	0.222	0.16	0.17
	p values	0.152	0.18	0.337	0.306
SXT	coefficient	0.266	0.32	0.12	0.177
	p values	0.106	0.05	0.472	0.287
CEF	coefficient	-0.161	-0.093	-0.162	-0.152
	p values	0.335	0.577	0.331	0.362
CAZ	coefficient	-0.058	-0.019	-0.065	-0.051
	p values	0.731	0.91	0.7	0.762
ENR	coefficient	-0.132	-0.084	-0.146	-0.017
	p values	0.431	0.618	0.381	0.918
OFX	coefficient	-0.103	-0.074	-0.169	-0.037
	p values	0.539	0.658	0.309	0.828
MEM	coefficient	0.266	-0.093	-0.007	-0.076
	p values	0.106	0.58	0.969	0.652
COL	coefficient	0.15	-0.098	0.293	-0.077
	p values	0.368	0.556	0.074	0.647

Table S3 The frequency of conjugative transfer of *mcr-1*-, *tet*(X4)- and *bla*$_{NDM-5}$-positive strains.

Strains	Frequency of conjugative transfer		
	Repeat 1	Repeat 2	Repeat 3
JX140	No	No	No
JX141	No	No	No
JX142	No	No	No
JX143	No	No	No
JX144	No	No	No
JX145	No	No	No
JX146	No	No	No
JX147	No	No	No
LS44	9×10^{-4}	3×10^{-3}	3.25×10^{-3}
LS45	1.18×10^{-2}	7.5×10^{-2}	2.73×10^{-2}
LS46	6.92×10^{-3}	1×10^{-3}	3.25×10^{-3}
LS47	2.22×10^{-2}	6.67×10^{-2}	1.5×10^{-2}
LS48	1.54×10^{-3}	2.86×10^{-3}	8.33×10^{-4}
LS49	1×10^{-2}	4×10^{-3}	5×10^{-3}
LS53	1.46×10^{-2}	5.56×10^{-3}	9.1×10^{-3}
LS54	1.38×10^{-2}	7.14×10^{-3}	1×10^{-2}
LS55	3.33×10^{-3}	9.10×10^{-3}	7.78×10^{-3}
LS56	5.71×10^{-3}	1.67×10^{-3}	2.86×10^{-3}
LS57	6.67×10^{-3}	7.14×10^{-3}	3.75×10^{-3}
NBJ303	No	No	No
JH65	No	No	No
JH74	No	No	No
LS62	2.25×10^{-3}	7.14×10^{-3}	7.73×10^{-3}
LS69	5.56×10^{-2}	2.08×10^{-2}	5.71×10^{-2}
LS80	4.55×10^{-3}	3.2×10^{-3}	3.18×10^{-3}
LS90	1.17×10^{-4}	1.36×10^{-5}	1.11×10^{-5}
TZ118	No	No	No
HZZ208	3.48×10^{-5}	5×10^{-5}	9.25×10^{-7}
JH51	4×10^{-5}	1.88×10^{-5}	2.73×10^{-5}
WZ22	No	No	No
HuZZ215	7.59×10^{-4}	1.4×10^{-3}	2×10^{-3}
